# The Relationship between Frontal QRS-T Angle and Vitamin D Deficiency

**DOI:** 10.3390/medicina60050776

**Published:** 2024-05-07

**Authors:** Fulya Avcı Demir, Gülsüm Bingöl, İbrahim Ersoy, Akif Arslan, Pınar Ersoy, Meltem Demir, Serkan Ünlü

**Affiliations:** 1 Department of Cardiology, Medical Park Hospital, 07160 Antalya, Turkey; dr.akifarslan@hotmail.com; 2 Department of Cardiology, Istinye University, 34010 Istanbul, Turkey; 3 Department of Cardiology, Istanbul Arel University, 34537 Istanbul, Turkey; bulut_gulsum@hotmail.com; 4 Department of Cardiology, Bahcelievler Memorial Hospital, 34180 Istanbul, Turkey; 5 Department of Cardiology, Kepez State Hospital, 07320 Antalya, Turkey; iersoytr@hotmail.com; 6 Department of Family Medicine, Akdeniz University, 07070 Antalya, Turkey; pinaraksoy07_86@hotmail.com; 7 Department of Biochemistry, Medikal Park Hospital, 07160 Antalya, Turkey; meldemir52@gmail.com; 8 Vocational School of Health Services, Antalya Bilim University, 07110 Antalya, Turkey; 9 Department of Cardiology, Gazi University Medical Faculty, 06570 Ankara, Turkey; unlu.serkan@gmail.com

**Keywords:** electrocardiography, frontal QRS-T angle, vitamin D

## Abstract

*Background and Objectives*: A deficiency in serum 25-hydroxyvitamin D levels is associated with a number of cardiovascular situations, such as high blood pressure, heart failure, atherosclerotic heart disease, and peripheral artery disease. The frontal QRS-T angle has recently been proposed as a marker of ventricular repolarization. A wider frontal QRS-T angle has been positively correlated with adverse cardiac events. The objective of our study was to examine the association between serum 25-hydroxyvitamin D level and the frontal QRS-T angle. *Materials and Methods*: A total of 173 consecutive patients aged 18–60 years undergoing routine cardiology check-up evaluation, and not receiving concurrent vitamin D treatment were included in the study. Patients were classified in three groups, depending on their vitamin D levels, and categorized as follows: Group 1—deficient (<20 ng/mL), Group 2—insufficient (20–29 ng/mL), or Group 3—optimal (≥30 ng/mL). The frontal QRS-T angle was determined using the automated reports generated by the electrocardiography machine. *Results*: The average age of participants was 45.8 (±12.2) years, and 55.5% of participants were female (*p* < 0.001). Individuals with low vitamin D concentrations exhibited a wider frontal QRS-T angle. It was determined that vitamin D level is an independent predictive factor for the frontal QRS-T angle. *Conclusions*: As the levels of 25-hydroxyvitamin D decrease, repolarization time assessed by frontal QRS-T angle is widened. Our findings indicate that lower concentrations of vitamin D may increase the susceptibility to ventricular arrhythmia.

## 1. Introduction

Vitamin D plays an important role in the absorption of phosphorus and calcium, in bone mineralization, and also in bone maturation [1]. It also contributes to the reabsorption of calcium and its analogs in the tubular space and is used in the treatment of various skin diseases, renal osteodystrophy, hypoparathyroidism, cancer, and even COVID-19 [2,3]. Beyond its well-known role in calcium balance and skeletal functions, the available evidence indicates that deficiency in serum vitamin D levels is a contributing factor for cardiovascular disease (CVD). A deficiency in vitamin D may result from inadequate dietary intake, abnormal metabolism, impaired absorption, or resistance to its effects [4]. The consequences of vitamin D deficiency differ depending on age. In children, rickets may present as osteomalacia or osteoporosis in adults [5]. Vitamin D also influences cardiac contractility and myocardial calcium hemostasis. Vitamin D deficiency/insufficiency has already been linked to the development of ion-channel disorders and autonomic dysfunction, which can cause cardiac arrhythmia [6]. Clinical and epidemiological studies have demonstrated a relation between low vitamin D concentrations and conditions such as atrial fibrillation, heart failure and coronary artery disease, recurrent cardiac events, and elevated risk of morbidity and mortality [7,8,9].

Ventricular repolarization (VR) parameters, including the QT dispersion (QTd), QT interval, corrected QT interval value (QTc) which can be detected by surface electrocardiography (ECG), have been frequently employed in studies aimed at predicting the risk of critical ventricular arrhythmias [10]. The more recent ECG parameter frontal QRS-T [f(QRS-T)] angle is an accepted indicator of cardiac electrical activity. It is the angle between the QRS wave that represents ventricular depolarization and the T wave that represents ventricular repolarization on a 12-lead surface ECG. The frontal method and the spatial method are two known methods for calculating the f(QRS-T) angle. Calculation with the frontal method is easy using the ECG report automatically generated by the ECG device. It is also significantly correlated with the spatial QRS-T angle, which is more complicated and so necessitates the use of advanced computer programs [11]. In optimal circumstances, the angle of the QRS complex and the T wave should be relatively narrow, indicating that depolarization and repolarization occur in unison. This observation also highlights the potential for cardiac electrical conduction and repolarization abnormalities that result from an increase in the f(QRS-T) angle. Previous studies have demonstrated that the f(QRS-T) angle may potentially serve as an independent prognostic marker in various cardiovascular pathologies [7,8,9,11]. A significant increase in the f(QRS-T) angle has already been linked to the occurrence of malign arrhythmia and sudden cardiac death [12,13]. There are studies on arrhythmia risk and vitamin D deficiency, but the possible relation between vitamin D deficiency and f(QRS-T) angle abnormalities remains unexplored [14]. The objective of our study is to determine the association between the f(QRS-T) angle and the severity of vitamin D deficiency/insufficiency.

## 2. Methods

### 2.1. Study Population

This is a retrospective, single-center, and cross-sectional study, aiming to identify the relation between the f(QRS-T) angle and serum vitamin D concentrations. The study includes 173 patients who were admitted to the cardiology outpatient clinic for routine check-ups between September 2023 and January 2024, consecutively. Participants aged between 18 and 65 years who had a standard 12-lead ECG recording and a serum 25-OH-vitamin D measurement on the same day were eligible for inclusion. The study participants were not concurrently on vitamin D treatment and were totally asymptomatic.

Participants with a history of chronic renal failure, heart failure, myocardial infarction, moderate/severe valvular disease, cerebrovascular event, infectious diseases, electrolyte abnormalities, malignancies, or any other condition known to alter cardiac conduction or repolarization were excluded. In addition, individuals with ECG artefacts, bundle-branch blocks, atrial fibrillation, or pacemaker rhythm were also excluded.

The study collected clinical and demographic data from patients, including their age, gender, and medical conditions such as hypercholesterolaemia, diabetes mellitus, and hypertension. Hypercholesterolaemia was defined as having a serum cholesterol level above 200 mg/dL or having received previous antihyperlipidaemic therapy. Diabetes mellitus was defined as a fasting blood glucose level above 126 mg/dL or the use of oral antidiabetic drugs, insulin therapy, or dietary therapy. Hypertension was diagnosed as having a blood pressure above 140/90 mmHG during two clinic visits or having received previous antihypertensive treatment. Current smokers were identified as individuals who reported smoking within the last 12 months. Additionally, the study calculated the patients’ body mass index (BMI) with the help of the formula: weight in kilograms divided by height in meters squared.

### 2.2. Laboratory Data

A series of laboratory tests were conducted on patients, including a complete blood count to determine hemogram, hematocrit, leukocyte, neutrophil, platelet, and mean platelet volume values; detailed renal and liver function tests; serum electrolyte levels; biochemical parameters such as C-reactive protein level (CRP); and thyroid function tests. In addition, 25-OH vitamin D levels were quantified from venous blood samples using the Architect 25-OH vitamin D analysis kit (Abbott Laboratories, Chicago, United States of America). Based on the latest guidelines, the patients enrolled in the study were classified in three groups according to their serum 25-OH vitamin D concentrations: Group 1, categorized as deficiency, i.e., vitamin D concentration was below 20 ng/mL; Group 2 was insufficiency, i.e., vitamin D concentration was between 20 and 29 ng/mL; and Group 3 was adequate, i.e., vitamin D concentration was 30 ng/mL or more [15,16].

### 2.3. Electrocardiography

A 12-lead standard ECG was administered to the patients, who were in the supine position after rest. The voltage was 10 mm/s, and the speed was 25 mm/s. The ECG was recorded using the Beneheart R12,199169 Mindray device. After scanned, ECGs were transferred to the computers of two different cardiologists, who were unaware of the patients’ identifiers, in order to prevent errors that may develop during sensitive measurements such as QT, corrected QT, QRS, Pd, Tp-e/QTc, Tp-e, and frontal QRS-t. ECGs were enlarged by 400% using the Adobe Photoshop program. The Tp-e interval, derived from a 12-lead surface ECG, refers to the distribution of ventricular repolarization throughout myocardial tissue and is expressed in terms of distance from the peak and the termination of the T wave [17]. The QT interval is the distance between the initiation of the QRS complex and the termination of the T wave and is an indicator of ventricular repolarization and depolarization. The QT interval is expressed as the distance from the initiation of the QRS complex to the termination of the T wave. Utilizing Bazett’s formula [QTc = QT√ (R-R interval)], the corrected QT (QTc) interval was calculated [18]. Subjects with discernible U waves were excluded to prevent measurement interference.

**Figure 1:**
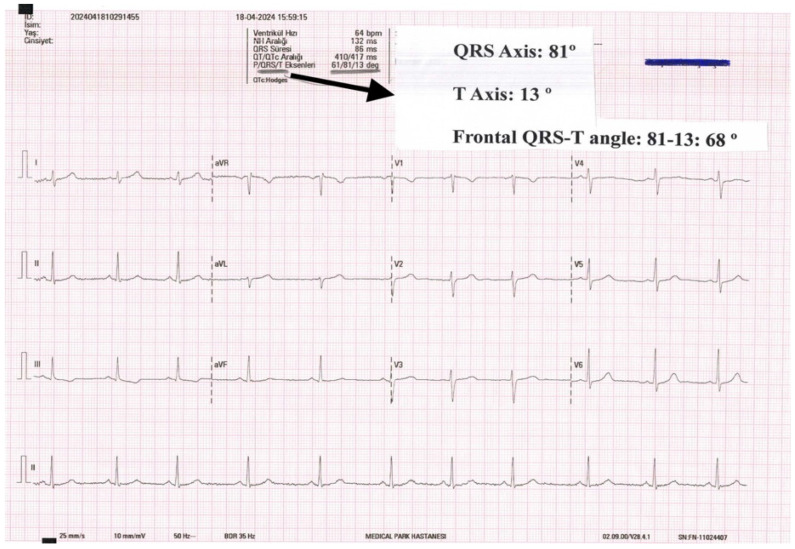
Method for calculating frontal QRS-T angle.

The f(QRS-T) angle is calculated via subtraction of the T axis from the QRS axis in the frontal plane (Figure 1). This represents the dimensional difference between the depolarization and repolarization vectors. In the event that the angle exceeds 180°, the calculation is performed by subtracting 360°. To avoid bias from subjective measurements, the f(QRS-T) angle is determined using the report automatically generated by the ECG machine. This method improves the accuracy and reliability of the f(QRS-T) angle calculation by using objective data from the machine.

### 2.4. Echocardiographic Assessment

Transthoracic echocardiography (TTE) examinations were conducted by two experienced cardiologists who were unaware of the other’s data. The examinations were conducted using a Vivid S5 ultrasound device (GE Vingmed Ultrasound AS, Horten, Norway) in accordance with the latest guideline recommendations [19]. The TTE evaluation consisted of standard M-mode assessment, two-dimensional measurements, and pulsed-wave and Color Doppler evaluation. Left ventricular ejection fraction (LVEF) was evaluated using the modified Simpson method. Pulsed-wave Doppler velocities included transmitral early- and late-diastolic peak-flow velocities and their ratio (Em, Am, and E/Am, respectively), which were obtained from the apical four-chamber window.

### 2.5. Statistical Analysis

Statistical analyses were carried out using JAMOVI, version 2.3.28, a third-generation open-source R-based software. For descriptive statistics, median and interquartile ranges (IQR) or mean and standard deviation (SD) were used. In the vitamin D subgroup, mean comparison was performed using an ANOVA test. Categorical variables were subjected to comparison using the chi-square test. For non-parametric variables, medians were analyzed with the Kruskal–Wallis test. Bonferroni corrections were used for post hoc analysis. Variables were analyzed using analytical methods to determine whether they were normally distributed.

Aiming to determine the association between f(QRS-T) angle and serum 25(OH)D levels, Pearson or Spearman correlation coefficients were employed to ascertain the optimal predictors of f(QRS-T) angle (i.e., vitamin D level, age, gender, coronary artery disease, hypertension, ejection fraction, diabetes mellitus, hemoglobin, thyroid hormone, vitamin B12 values, BMI, and glucose), using the multivariate linear regression input method. A *p*-value lower than 0.05 was accepted as statistically significant.

### 2.6. Ethical Considerations

This study was approved by the Ethics Committee of Memorial Hospital, Istanbul, Turkey on 5 January 2023, with approval code number 84. The study was conducted in line with the principles of Declaration of Helsinki and Good Clinical Practice, with the aim of respecting the rights and dignity of all parties involved. All participants were provided with detailed information about the purpose and the protocol of the study, after which verbal informed consent was taken from all of them.

## 3. Results

Table 1 summarizes the baseline demographic, clinical, electrocardiographic (ECG), and echocardiographic characteristics of the study population for various vitamin D levels. In total, 173 participants were included in the study, and 55.5% of them were female (*p* < 0.001). The mean age of the study group was 45.8 (±12.2) years. Among participants, there were not any significant differences in body mass index (BMI); sex; high blood pressure; HDL cholesterol, LDL cholesterol, and triglyceride levels; glucose levels; smoking status; known coronary artery disease; renal kidney; and thyroid functions among various vitamin D level groups (*p* > 0.05 for all). The ECG parameters, including QRS duration, QT interval, QTc interval dispersion, *p* wave duration, interval from peak of the T wave to its termination, and the ratio of Tp-e to QTc, remained comparable across all subgroups (*p* > 0.05 for all). However, the f(QRS-T) angle demonstrated a tendency to increase with elevated levels of vitamin D level (*p* < 0.001).

Vitamin D levels did not significantly impact most echocardiographic parameters such as left atrial and ventricular dimensions, left ventricular wall thickness, and pulsed-wave Doppler findings. Only the f(QRS-T) angle showed a notable association with vitamin D levels.

Univariate linear regression demonstrated a statistically significant association between the f(QRS-T) angle and several variables, including hemoglobin level [unstandardized regression coefficient (B): 0.155, 95% Confidence interval (CI): 0.297 −0.006, *p* = 0.042], female gender (B: 0.160, 95% CI: 0.021 0.311, *p* = 0.036), and 25-OH-vitamin D (B: −0.316, 95% CI: −0.621 −0.248, *p* < 0.001). No significant difference was identified in LVEF values between the groups. Multivariate analysis identified that LV EF [standardized regression coefficient (β): 1.379, 95% CI: 0.126 2.632, *p* = 0.031] and 25-OH-vitamin D levels (β: −0.422, 95% CI: −0.602–0.243, *p* < 0.001) were independent predictors of the f(QRS-T) angle. These findings are summarized in Table 2.

**Figure 2:**
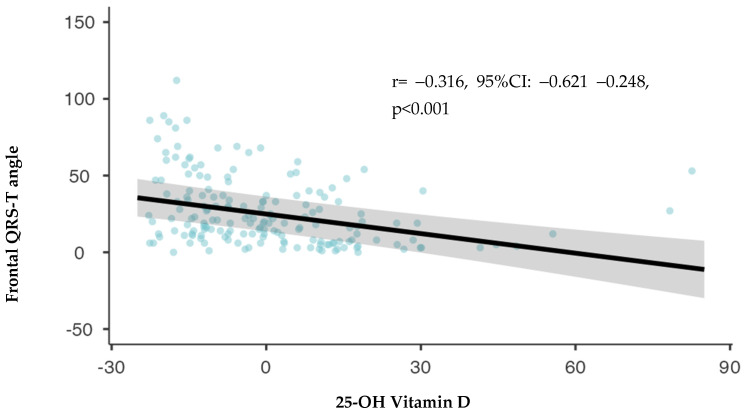
Correlation analysis between frontal QRS-T axis and 25–OH vitamin D levels.

Furthermore, a strong association between the f(QRS-T) angle and vitamin D is seen in Figure 2 (r= −0.316, *p* < 0.001).

## 4. Discussion

This study demonstrates a strong correlation between vitamin D deficiency/insufficiency and prolonged f(QRS-T) angle in patients. This discovery implies that vitamin D may directly affect the spatial relationship between cardiac depolarization and repolarization, as indicated by the f(QRS)T angle. Our findings align with previous research that links vitamin D levels to cardiovascular complications [20]. According to our knowledge, no studies have been conducted thus far that utilize this novel parameter to assess the relationship between f(QRS)T angle and serum 25-OH vitamin D levels.

Vitamin D and its receptor exert a significant influence on organ function, particularly in the cardiovascular system, musculoskeletal system, central nervous system, and hepatorenal system. The 1α-hydroxylase enzyme is secreted by smooth muscle cells, vascular endothelial cells, fibroblasts, and cardiomyocytes, and it increases the level of serum-active vitamin D and its binding receptors [21]. Vitamin D exerts a significant influence on the cardiovascular system, including the prevention of proliferation and hypertrophy in vascular smooth muscle cells and myocytes, the suppression of the renin–angiotensin–aldosterone system (RAAS), and the enhanced secretion of vascular endothelial growth factor. As a consequence of these effects, its role in myocardial contractility and calcium balance is also of great importance. A deficiency in vitamin D has been demonstrated to be correlative with the development of a number of cardiovascular diseases, including hypertension, atherosclerotic coronary and peripheral vascular disease, and systolic and diastolic dysfunction [22]. A study by Szabo et al. evaluated the relationship between biochemical markers such as vitamin D, uric acid, and albumin, whose roles in heart failure have been previously defined, and the systolic function of the left ventricle in patients with reduced and mildly reduced ejection fraction. The study identified vitamin D as an independently predictive factor of LVEF in heart failure patients and highlighted its potential interaction with uric acid and albumin [23]. Furthermore, vitamin D deficiency has been demonstrated to precipitate sudden cardiac death (SCD) and fatal cardiac arrhythmia through the mechanisms of ionic heart disease, channelopathies, and autonomic dysfunction [14,22].

The f(QRS-T) angle has been identified as a robust and independent predictor of cardiac morbidity and mortality, including sudden cardiac death. A wider angle indicates potential alterations in myocardial ion channel function and abnormal regulation of ventricular repolarization [24,25]. The f(QRS-T) angle is a more specific, more sensitive, and more practical marker of myocardial repolarization than the QT interval, which also indicates abnormalities in myocardial depolarization. The advantages of f(QRS-T) angle are highlighted by the complexities in measuring QT interval and inter-observer variability [26]. Additionally, QT dispersion does not accurately reflect the regional heterogeneity in cardiac repolarization [27].

Our results align with other studies that investigate novel repolarization markers. For instance, one research work indicates an increased mortality rate in patients with Brugada syndrome if the Tp-e interval is prolonged [17]. Given the fact that the Tp-e interval is susceptible to alterations in the patient’s heart rate and body mass index, the Tp-e/QTc ratio is regarded as a more reliable method for evaluating repolarization than the Tp-e interval [28]. Nevertheless, the accurate calculation of the Tp-e/QTc ratio is challenging and necessitates the use of advanced software programs, in addition to rulers and magnifying glasses as specific tools for this purpose. Bagrul et al. observed that children with vitamin D deficiency exhibited prolonged Tp-e intervals, in addition to elevated Tp-e/QTc and Tp-e/JTpeak ratios [29].

While our findings reveal a correlation between f(QRS-T) angle and vitamin D levels, they did not demonstrate an association with the Tpeak-to-Tend interval (Tp-e), Tp-e/QT and Tp-e/QTc ratios, or QT dispersion (QTd). This highlights the complex interactions within cardiovascular electrophysiology and suggests that other variables may influence vitamin D’s impact [30]. Overall, f(QRS-T) angle serves as a valuable and practically measurable tool for identifying abnormal ventricular repolarization and predicting arrhythmic risk [24].

Although our study design provides valuable information, some limitations need to be considered. The cross-sectional and retrospective design of our study prevents the possibility of establishing causal divisions. As the patients included in the study were asymptomatic and only seen once, there are no data available about symptoms like palpitation, ECG findings or 24 h ECG Holter recordings during follow-up.

Furthermore, the absence of any physical examination, direct radiography, or bone densitometry regarding the potential clinical conditions that may develop in the musculoskeletal system in vitamin D deficiency precludes any comment on the long-term effects of vitamin D deficiency based on the study data.

Consequently, a number of observational studies conducted on this subject in the future are required to confirm the long-term effects of serum 25-OH vitamin D levels on f(QRS-T) angle. Additionally, these long-term follow-up studies will be invaluable in observing the symptoms that may develop in patients over time and the cardiac and musculoskeletal diagnoses that may be detected. The results of these studies will inform our clinical practice. Larger sample sizes would increase statistical power and allow us to detect more subtle effects that are related to vitamin D status. Future research should examine the interaction between vitamin D and other factors that influence cardiac conduction. Although we include several covariates in our multivariate models, we can no longer rule out the possibility of confounding.

## 5. Conclusions

Our findings indicate that the f(QRS)T angle is significantly widened in patients with vitamin D deficiency or insufficiency. This result gains greater clinical significance when combined with studies showing that vitamin D deficiency is an independently predictive factor of increased cardiovascular morbidity and mortality. Clinicians should keep these findings in mind when monitoring vitamin D levels and planning treatment. Further research is needed to substantiate these findings and elucidate the underlying mechanisms.

## Figures and Tables

**Table 1:** Baseline demographic and clinical features of the study population.

	Group 1 * (n = 69)	Group 2 * (n = 41)	Group 3 * (n = 63)	Total (n = 173)	*p*-Value ^1^
**Age (years) (±SD)**	46.1 ± 11.6	48.4 ± 13.4	45.3 ± 12.2	45.8 ± 12.2	0.925 ^2^
**Sex (female), n (%)**	41 (58.6)	16 (41.0)	39 (60.9)	96 (55.5)	0.114 ^1^
**BMI, kg/m^2^ (±SD)** **Diabetes Mellitus, n (%)**	26.6 ± 5.1 8 (11.6)	25.6 ± 4.8 8 (19.5)	26.2 ± 4.2 7 (11.1)	26.2 ± 4.7 23 (13.3)	0.548 ^2^ 0.405 ^1^
**Hypertension, n (%)** **Family History, n (%)** **Smoking, n (%)**	11 (15.9) 19 (27.5) 27 (39.1)	5 (12.2) 2 (4.9) 9 (21.9)	12 (18.8) 16 (26.6) 29 (46.0)	28 (16.2) 37 (21.4) 65 (37.6)	0.649 ^1^ 0.012 ^1^ 0.044 ^1^
**Dyslipidemia, n (%)**	2 (2.9)	3 (7.3)	7 (11.1)	12.0 (6.9)	0.180 ^1^
**CAD, n (%)**	1 (1.4)	2 (4.9)	2.0 (3.1)	5.0 (2.9)	0.625 ^1^
**Glucose, g/dL (±SD)** **AST, U/L (±SD)**	110.2 ± 44.8 19.7 (11.0–44.0)	98.7 ± 16.1 20.9 (10.0–93.0)	100.6 ± 31.8 21.1 (6.0–130.0)	103.9 ± 35.3 20.5 (6.0–130.0)	0.161 ^2^ 0.821 ^2^
**Creatinine, mg/dL (** **±SD** **)**	0.76 ± 0.15	0.82 ± 0.18	0.79 ±0.17	0.8 ± 0.2	0.048 ^2^
**ALT, U/L (** **±SD** **)**	25.9 (10.0–96.0)	21.5 (8.0–233.0)	26.3(6.0–301.0)	25.0 (6.0–301.0)	0.707 ^2^
**TSH, mU/L (** **IQR** **)**	2.2 (1.2–2.5)	2.0 (1.2–2.9)	1.8 (1.1–2.5)	1.9 (1.2–2.5)	0.772 ^3^
**Hemoglobin, g/dL (** **±SD** **)**	14.1 ± 1.8	14.5 ± 1.6	14.4 ± 1.5	14.3 ± 1.7	0.417 ^2^
**EF, % (** **±SD** **)**	62.3 ± 2.5	62.5 ± 2.5	62.6 ± 2.5	62.5 ± 2.5	0.896
**LA, mm (** **±SD** **)**	35.3 ± 3.9	35.3 ± 4.4	35.4 ± 4.5	35.3 ± 4.3	0.979
**LVDD, mm (** **±SD** **)**	46.2 ± 3.8	45.7 ± 4.0	45.8 ± 3.8	45.9 ± 3.7	0.526
**IVS, mm** **(** **±SD** **)**	9.8 ± 1.6	9.7 ± 2.3	9.6 ± 2.2	9.7 ± 2.0	0.475
**PW, mm** **(** **±SD** **)**	9.8 ± 1.6	9.4 ± 2.7	9.6 ± 2.3	9.7 ± 2.2	0.359
**Mitral E/A, m/s** **(** **±SD** **)**	1.27 ± 0.46	1.30 ± 0.37	1.33 ± 0.38	1.3 ± 0.4	0.405
**QTc, ms (** **±** **SD)**	39.1 ±6.5	40.5 ± 8.2	40.7 ± 6.2	40.0 ± 6.9	0.206 ^1^
**QTcd, ms (** **±** **SD)**	40.0 ±6.8	44.2 ± 8.7	44.2 ± 6.6	43.8 ± 7.3	0.181 ^1^
**Pd, ms (** **±** **SD)**	37.3 ±6.1	36.8 ± 7.1	38.4 ± 7.1	37.6 ± 6.7	0.544 ^1^
**Tp-e, ms (** **±** **SD)**	74.4 ±11.8	74.6 ± 10.5	74.5 ± 10.7	74.5 ± 11.1	0.897 ^1^
**Tp-e/QTc, ms (IQR)**	0.19 (0.16–0.22)	0.18 (0.17-.0.20)	0.19 (0.17–0.20)	0.19 (0.16–0.20)	0.723 ^3^
**QTc, ms (** **±** **SD)**	403.6 ±19.8	409.8 ±17.8	405.7 ± 20.0	405.8 ±19.5	0.588 ^1^
**f(QRS-T) angle, °, IQR)**	28 (14.5–53)	20 (12.5–31.5)	8 (4–27)	19 (8.5–37)	<0.001 ^3^

^1^:Pearson’s chi-square test, ^2^:Lineer model ANOVA, ^3^:Kruskal–Wallis test, SD: Standard Deviation, ALT: Alanine Aminotransferase, AST: Aspartate Aminotransferase, BMI: Body Mass Index, CAD: Coronary Artery Disease, f(QRS-T): Frontal QRS-T angle, HDL: High-Density Lipoprotein Cholesterol, IQR: Interquartile Range, LDL: Low-Density Lipoprotein Cholesterol, Pd: P-wave duration, QRS: QRS duration, QTc: Corrected QT interval QTcd: Corrected QT interval dispersion, TSH: Thyroid Stimulating Hormone, Tp-e: Interval from the peak to the termination of the T wave, Tp-e/QTc): Ratio of Tp-e to corrected QT interval, *: Group 1 = Vitamin D ≤ 20, Group 2 = 20 < Vitamin D < 29, Group 3 = 30 ≤ Vitamin D.

**Table 2:** Univariate and multivariate linear regression analysis of predictors for frontal QRS-T angle.

	Univariate				Multivariate			
Variables			95% Confidence Interval				95% Confidence Interval	
	B	*p*-Value	Lower	Upper	Β *	*p*-Value	Lower	Upper
**Sex (Female)**	0.160	**0.036**	0.021	0.311	-	-	-	-
**Age**	0.168	**0.027**	0.019	0.390	-	-	-	-
**BMI**	0.121	0.113	−0.029	0.265	-	-	-	-
**Hemoglobin**	0.155	**0.042**	−0.297	−0.006	-	-	-	-
**Hypertension**	0.016	0.029	−0.021	0.272	-	-	-	-
**Diabetes**	0.038	0.531	−0.102	0.196	-	-	-	-
**LV EF**	0.128	0.092	−0.021	0.272	1.379	**0.031**	0.126	2.632
**Glucose**	0.062	0.420	−0.088	0.290	-	-	-	-
**B12**	0.008	0.485	−0.004	0.022	-	-	-	-
**TSH**	0.626	0.262	−1.288	2.642	-	-	-	-
**25-OH, Vitamin D**	−0.316	**<0.001**	−0.621	−0.248	−0.422	**<0.001**	−0.602	−0.243
**CAD**	0.055	0.750	−0.102	0.173	-	-	-	-

B: unstandardized regression coefficient, CAD: Coronary artery disease, β: standardized regression coefficient, TSH: Thyroid-stimulating hormone, BMI: Body mass index, LV EF: Left ventricular ejection fraction. *: F:2.347, R^2^:0.230, *p* < 0.001.

## Data Availability

The original contributions presented in the study are included in the article further inquiries can be directed to the corresponding author.
